# Iodine and selenium intakes and status and thyroid function in midlife women with low bread intakes in New Zealand

**DOI:** 10.1111/1747-0080.70025

**Published:** 2025-06-18

**Authors:** Jaime Berger, Jacqueline Finlayson, Pamela R. von Hurst, Louise Brough

**Affiliations:** ^1^ School of Sport, Exercise and Nutrition College of Health, Massey University Auckland New Zealand; ^2^ School of Food Technology and Natural Sciences College of Sciences, Massey University Palmerston North New Zealand

**Keywords:** iodine, midlife women, selenium, thyroid

## Abstract

**Aims:**

Iodine and selenium are important nutrients for thyroid function; however, the New Zealand food supply is generally low in both minerals. Bread can be a good source of these minerals; although the popularity of lower carbohydrate diets means some people avoid bread. This study aimed to investigate the effect of low bread intakes on iodine and selenium intakes and status, and thyroid function in mid‐life women in New Zealand.

**Methods:**

Self‐selecting women (*n* = 46), aged 40–63 years, with a mean daily intake of 1.6 ± 1.5 slices of fortified commercial bread, were recruited into a cross‐sectional study in Auckland, New Zealand. Assessment of iodine and selenium intake was via a 3‐day diet diary. Iodine and selenium concentrations were measured in 24‐h urine samples and selenium concentrations in plasma using inductively coupled plasma mass spectrometry. Thyroid hormones including triiodothyronine and thyroxine were also determined.

**Results:**

Median urinary iodine concentration was 49 (35, 78; 25, 75 centile) μg/L indicating iodine deficiency. Of plasma samples measured, 32% had selenium concentrations below 110 μg/L, suggesting inadequacy. Data suggested at least 40% of participants had low intakes of both nutrients. Only two participants had impaired thyroid function; however, plasma selenium concentrations predicted the ratio of triiodothyronine to thyroxine (*p* = 0.038).

**Conclusions:**

A high prevalence of inadequate selenium and iodine intake was observed in women with low bread intakes. Those with low bread intakes need to ensure they consume alternative sources such as dairy, fish and seafood, eggs, meat, other grains, and nuts.

## 1 INTRODUCTION

Iodine and selenium are essential for the biosynthesis of thyroid hormones, which control growth, metabolism, and cognitive development in humans.^1^ Iodine is a component of the thyroid hormones, thyroxine (T4) and triiodothyronine (T3), and the deiodinases that convert inactive T4 to the active T3 are selenoproteins.^2^, ^3^ Further, the selenoprotein glutathione peroxidase (GPx) protects the thyroid gland against hydrogen peroxide generated during thyroid hormone production.^4^ Iodine deficiency can result in an accumulation of hydrogen peroxide, leading to further damage to the thyroid gland.^1^ Consequently, there is potential for iodine and selenium deficiency in adults to result in hypothyroidism and goitre.^5^ In New Zealand, thyroid disease is more common in women (5%) than men (1%),^6^ and for women older than 50 years, the prevalence increases to 7%–14%.^7^

New Zealand has low levels of both iodine and selenium in the food supply, which puts the population at risk for deficiency.^8^ New Zealand has a long history of inadequate iodine intake; from the late 1800s to the early 1900s, iodine deficiency (ID) was significant, and endemic goitre and very low urinary iodine concentrations prevailed. The iodisation of salt, introduced in 1924, reduced ID and almost eradicated endemic goitre.^9^ The use of iodophors (iodine‐based cleansers) by the dairy industry also helped to raise population iodine intake, and in the 1970s, New Zealand had low goitre rates. However, ceasing the use of iodophors in the dairy industry, alongside changes in dietary patterns and other factors, saw the re‐emergence of ID in the 1990s. In 2009, food regulations made it mandatory for all commercially made bread (except organic) to be manufactured with iodised salt in an attempt to raise population intake.^10^

Bread is the largest single contributor of selenium to the New Zealand diet.^11^ In the North Island, bread is made predominantly using imported Australian wheat, which is a rich source of selenium.^4^, ^12^ Therefore, habitual bread consumption is key to maintaining a good dietary intake of both essential minerals.

In both Australia and New Zealand, the Estimated Average Requirement (EAR) and Recommended Dietary Intake (RDI) for adult women for iodine are 100 and 150 μg/day and for selenium are 50 and 60 μg/day, respectively.^13^ The 2009 New Zealand Total Diet Survey (NZTDS) reported the mean dietary intake of iodine of adult females to be 63 μg/day, well below the EAR, suggesting inadequate intake for more than half the group.^14^ The most recent NZTDS (2016)^15^ reported the mean dietary intake of iodine for adult females at 100 μg/day, which is a marked improvement from the earlier 2009 NZTDS, reflecting the mandatory fortification of bread. Similarly, while the 2009 NZTDS reported mean dietary intakes of selenium of adult females to be 56 μg/day, the most recent (2016) NZTDS suggested that average selenium intakes are now adequate at 68 μg/day.^14^, ^15^ The 2016 NZTDS attributes the increases in selenium intake to increased selenium concentrations in the food supply rather than increased consumption of selenium‐rich foods such as meat.^15^ However, the NZTDS estimates intake based on standard diets and may not reflect actual intake.

The reliance on bread for both iodine and selenium intake is of concern due to current trends for reducing carbohydrate intake. High fat, low carbohydrate diets have increased in popularity, leading to reduced bread consumption. Mid‐life women in New Zealand have significantly reduced their bread intake over the past two decades from a median of 3–4 slices per day in 1997 down to a median of 1.8 slices per day in recent studies.^16^, ^17^ New Zealanders who choose to avoid commercially prepared bread therefore could be at an increased risk of both iodine and selenium deficiency, if not obtaining these nutrients from other food sources.

This study aimed to investigate the effect of low bread intakes on both iodine and selenium intakes and status, and association with thyroid function in mid‐life women in New Zealand.

## 2 METHODS

Women (*n* = 46) were recruited into this cross‐sectional study (WOMBI; Women, bread and iodine) via the use of posters and social media releases in Auckland, New Zealand from April to August 2017. In 2020, consent was requested to have previously collected blood and urine samples measured for selenium and thyroid hormones, resulting in this data for a subset of 37 participants. This study was performed in line with the principles of the Declaration of Helsinki. Ethical approval was granted by Massey University Human Ethics Committee Southern A (application numbers 16/52 and 20/28). This research was carried out in accordance with the STROBE guidelines for observational studies.

Eligible participants visited the nutrition research unit at Massey University, Albany, Auckland, New Zealand for a 30–45‐min session to facilitate data collection. Participants provided written, informed consent and were women aged 40–65 years who reported consuming less than one slice of bread per day. Exclusion criteria were eating more than one slice of bread per day, smoking, having thyroid disease or using thyroid medication, taking supplements containing ≥10 μg/day iodine or using kelp salt (high iodine content) daily. Information was also collected about the frequency of bread consumption and the type of bread consumed over the previous 2 months. None of the participants were taking medications that alter iodine excretion (i.e., loop diuretics or amiodarone).

Each participant completed a 3‐day diet diary including two weekdays and one weekend day. Participants were asked to estimate portion size and report brand, method of cooking, and dietary supplements. Clear instructions and examples were provided. Data was entered into Foodworks professional package for Windows version 10 (Xyris software [Australia] Pty Ltd., 2019) using the standard database for New Zealand (NZ foodfiles 2016) to determine mean daily iodine and selenium intake. The iodine content of all commercial bread has not been determined since the mandatory addition of iodised salt to bread. However, iodine concentrations have been estimated by the Ministry for Primary Industries for categories of bread (e.g., white, fibre white, fruited, mixed grain, etc.)^18^ and these values were entered into Foodworks. Participants who reported using iodised salt daily (*n* = 6) had 1 g of iodised salt (49.3 μg iodine) per day added to their total iodine intake (estimated as an average intake in a previous New Zealand study).^19^

Using the 3‐day diet diary data, the amount of selenium and iodine from each food item consumed was determined. Each food was assigned to a food group known to contain iodine and/or selenium (dairy, meat, fish and seafood, eggs, bread, grains [excluding bread], fruit, vegetables, legumes, nuts and seeds, seaweed, chocolate, and drinks and water) and the amount from each food was summed. The proportion of each nutrient from each food group was determined as a percentage. Other foods typically with percentages <0.5% were grouped as ‘Other’. Discretionary iodised salt was not included in this calculation due to difficulties in measuring actual intake.

Participants were provided with equipment and instructions to collect a 24‐h urine sample. Urine samples were sent to an accredited commercial laboratory, Hills Laboratory (Hamilton, NZ) for analysis of both selenium and iodine using inductively coupled plasma mass spectrometry (ICPMS). Quality control measures included analysis of blanks, analytical repeats, and certified reference samples to ensure accuracy and precision. On every run, calibration standards and checks were undertaken, with the limit of detection at 0.002 mg/kg for selenium and 0.001 mg/kg for iodine. Batches of 25 samples were analysed together with an external reference standard (Seronorm Trace Elements Urine, L‐2, Norway) giving a mean (SD) iodine concentration of 286 (12) μg/L (published value: 297 μg/L, 95% confidence interval 237–356) with a coefficient of variance (CV) of 4.2% (*n* = 19) and mean (SD) selenium concentration of 74 (5) μg/L (published value 71.7 μg/L, 95% confidence interval 57.3–8.61 μg/L) with a CV of 6.9% (*n* = 16).

Daily intakes of iodine and selenium intakes were estimated from 3‐day diet diaries and also by extrapolation from 24‐h urinary iodine and selenium excretion based on the amounts excreted via urine (90% for iodine^20^ and 55% for selenium^12^).

A non‐fasting sample of blood (20 mL) was taken from each participant's antecubital fossa by a qualified phlebotomist at the research unit. Blood samples were processed, and plasma and serum samples were stored at −80°C before sending to an accredited commercial laboratory; Canterbury Health Laboratory (Christchurch, NZ). Plasma selenium concentration was determined by ICPMS. Free T3, free T4, thyroid stimulating hormone (TSH), thyroglobulin (Tg), thyroglobulin antibodies (TgAb), and thyroid peroxidase antibody (TPOAb) were measured via chemiluminescent microparticle immunoassay (CMIA). Reference ranges for euthyroid are: TSH, 0.40–4.0 mIU/L; free T4, 10–24 pmol/L; and free T3, 2.5–6.0 pmol/L. These reference ranges were used to determine the prevalence of thyroid dysfunction, including subclinical hypothyroidism (TSH >4.00 mIU/L and free T4 between 10 and 24), overt hypothyroidism (TSH >4 mIU/L and free T4 < 10 pmol/L), subclinical hyperthyroidism (TSH <0.4 mIU/L and free T4 between 10 and 24 pmol/L) and overt hyperthyroidism (TSH <0.4 mIU/L, free T4 >24 pmol/L and free T3 >6.0 pmol/L). For participants with TgAb concentration greater than 115 IU/mL, Tg values were discounted due to a high likelihood of it being artificially lowered by TgAb. TPOAb concentrations above 10 IU/mL indicate a potential autoimmune disorder.

Statistical analysis was performed using SPSS Version 24 for Windows (IBM Corp, 2016). Data were tested for normality using the Shapiro–Wilk test, recommended when *n* < 50. Normally distributed data were expressed as mean ± standard deviation (SD) and median (25, 75 centile; Q1, Q3), and non‐normal data were expressed as median (Q1, Q3). Correlations were tested using Spearman's correlation coefficient as much of the data was not normally distributed. Multiple linear regression was used to determine predictors of the T3:T4 ratio. Predictor variables included plasma selenium and urinary iodine excretion over 24 h, which were entered into the model simultaneously. Age and BMI were not included as they are not known to influence iodine or selenium status, and the sample was relatively homogeneous. Statistical significance was set at *p* < 0.05.

## 3 RESULTS

Participants were aged between 40 and 63 years, with a mean age of 50.8 (±6.2) years and mean height 163.8 (±6.1). Median body weight was 70.5 (62.4, 81.4) kg, and median body mass index (BMI) was 25.7 (24.5, 28.8) kg/m^2^, with BMI ranging between 19.3 and 47.6 kg/m^2^. All participants consumed, on average, less than one slice of bread per day. Mean bread consumption was 1.6 ± 1.5 serves of bread (serve is one slice of bread or one bread roll) per week, with a range of 0–5 servings per week.

Median urinary iodine concentration (mUIC) was 49 (35, 78) μg/L, below the WHO population threshold of 100 μg/L and also below the alternative cut‐off for adults of 60–70 μg/L,^21^ suggesting deficiency (Table 1). Median iodine intakes were 63 (47, 84) μg/day based on 3‐day diet diaries and 120 (81, 171) based on urinary iodine excretion, with 82% and 41% below the EAR (100 μg/day), respectively.

**TABLE 1:** Iodine and selenium intake and status in midlife women from Auckland, New Zealand.

Biomarker	Assessment method	*n*	Mean (SD)	Median (25, 75 centile)	*n* (%) participants below EAR^a^
Iodine intake (μg/day)	3DDD^b^, ^e^	45	‐	63 (47, 84)	37 (82%)
	Urine^d^, ^e^	46	‐	120 (82, 171)	19 (41%)
Selenium intake (μg/d)	3DDD^b^, ^e^	45	‐	52 (43, 75)	18 (40%)
	Urine^e^	37	‐	51 (38, 72)	18 (49%)
Iodine status	UIC (μg/L)^c^, ^e^	46	‐	49 (35, 78)	‐
	UIE (μg/d)^d^, ^e^	46	‐	108 (74, 154)	‐
Selenium status	Plasma selenium concentration (μg/L)	37	120.5 (19.4)	119.3 (107.4, 132.3)	‐

^a^ EAR = Estimated Average Requirement is 100 μg/day for iodine and 50 μg/day for selenium for female adults.

^b^ 3DDD—3 day diet diary.

^c^ UIC = urinary iodine concentration (μg/L).

^d^ UIE = urinary iodine excretion (μg/day).

^e^ Data not normally distributed.

Estimated median selenium intake was 52 (43, 75) μg/day based on 3‐day diet diaries and 51 (38, 72) μg/day based on urinary excretion, with 40% and 49% below the EAR (50 μg/day), respectively (Table 1). Dietary iodine and selenium intake were moderately correlated based on 3‐day diet diaries (*p* = 0.009, *r* = 0.383) and urinary excretion (*p* < 0.001, *r* = 0.668). Thirty‐three percent of participants had intakes for both iodine and selenium below the EAR based on 3‐day diet diaries (15 out of 45 participants) and urinary excretion (*n* = 12 out of 37 participants).

Median plasma selenium concentration for this study was 119.3 (107.4, 132.3) μg/L and all participants were above 65–71 μg/L, required for optimal deiodinase function.^22^, ^23^ However, three participants had plasma concentrations lower than 95 μg/L required for saturation of GPx activity^24^ and 12 had concentrations lower than 110 μg/L, proposed for maximum expression of selenoprotein P^25^ indicating inadequacy for some participants.

Analysis of the 3‐day diet diaries (excluding discretionary iodised salt) showed dairy to contribute the most iodine at 23.6% (Table 2) followed by fish and seafood (21.7%) and eggs (13.8%). Fish and seafood was also the most significant contributor to selenium intake at 35.3%, followed by nuts and seeds (17.2%) and meat (12.7%). The predominant contributor to the nuts and seeds food group was Brazil nuts due to their high selenium content. However, while participants were eating less than one slice of bread per day, 3‐day diet diaries revealed that participants were still consuming wheat and other grains contributing 9.0% of total selenium intake. Eggs were another significant contributor of selenium at 7.6%. As expected, bread made only a low contribution to iodine (6.1%) and selenium (2.7%).

**TABLE 2:** Percentage contribution of foods to iodine and selenium for midlife women (*n* = 45) based on a 3‐day diet diary (excluding discretionary iodised salt).

Food	Iodine (%)	Selenium (%)
Dairy	23.6	4.8
Fish and seafood	21.7	35.3
Eggs	13.8	7.6
Bread	6.1	2.7
Drinks and water	5.9	0.9
Vegetables	5.9	6.6
Seaweed	5.0	0.1
Meat	4.6	12.7
Grains (excluding bread)	4.5	9.0
Nuts and seeds	3.2	17.2
Chocolate	1.9	0.6
Fruit	1.0	0.7
Legumes	0.8	1.6
Other	2.9	0.5

Based on the 3‐day diet diaries, the mean percentage of energy provided by macronutrients was 33.5% carbohydrate, 18.7% protein, 38.3% total fat, and 14.0% saturated fat (Table 3). The acceptable macronutrient distribution ranges for carbohydrate, protein, total fat, and saturated fat are 45%–65%, 15%–25%, 20%–35%, and <10% of energy, respectively. Compared to the recommendations, participants were consuming a diet that is lower in carbohydrate and higher in both total and saturated fat. Mean daily intake of fibre was 23.9 g, with 67.0% having intakes below the Suggested Dietary Target (28 g/day). Compared to the most recent national survey (2008/09 Annual Nutrition Survey),^11^ participants were on average consuming less carbohydrate and sugar, but more fibre.

**TABLE 3:** Estimated macronutrient intakes in midlife women from 3‐day diet diaries, compared to recommendations and estimated intakes for women from the 2008/09 Annual Nutrition Survey.

Macronutrient	Mean (SD) intake (% of total energy)	AMDR (% of total energy)	Mean intakes from ANS 2008/09^b^ (% total energy)
Carbohydrate	33.5 (9.0)	45–65	47.1
Protein	18.7 (5.5)	15–25	16.5
Total fat	38.3 (8.8)	20–35	33.8
Saturated fat	14.0 (4.8)	<10	13.1
Monounsaturated^a^	25.3 (19.9, 31.0)	‐	12.3
Polyunsaturated^a^	10.3 (6.8, 15.8)	‐	4.9

	Mean intake (g/day)	Suggested dietary target (g/day)	Mean intakes from ANS 2008/09^b^ (g/day)
Fibre	23.9 (9.6)	28	17.9
Sugar^a^	69.9 (50.8, 96.6)	‐	96 (91101)

Abbreviation: AMDR, acceptable macronutrient distribution range; ANS, Annual Nutrition Survey.

^a^ Median (Q1, Q3) as data not normally distributed.

^b^ ANS 2008/09 = New Zealand Adult Nutrition Survey.^11^

Only two participants (5%) had low levels of TSH (with normal free T4) and therefore are considered to have subclinical hyperthyroidism (Table 4). Most participants (*n* = 35) had thyroid markers indicating euthyroidism. Four participants (11%) had a TPOAb concentration greater than 10 IU/mL indicating a potential autoimmune disorder. Four participants had a TgAb concentration greater than 115 IU/mL and therefore their Tg concentration was disregarded due to the likelihood of it being artificially lowered by the Tg antibodies.

**TABLE 4:** Thyroid function markers and prevalence of thyroid dysfunction in midlife women (*n* = 37).

Biomarker	Median (25, 75 centile)
TSH (mIU/L)	1.05 (0.8, 1.8)
Free T4 (pmol/L)	12.5 (12, 13)
Free T3 (pmol/L)	4.7 (4.4, 4.9)
Free T3:T4 ratio	0.37 (0.35, 0.40)
Tg (μg/L) (*n* = 33)^f^	16.3 (9.0, 22.7)

	*n* (%)
TPOAb+ (>10 IU/mL)^a^	4 (11)
Subclinical hypothyroidism^b^	0
Overt hypothyroidism^c^	0
Subclinical hyperthyroidism^d^	2 (5)
Overt hyperthyroidism^e^	0

*Note*: Reference ranges for euthyroidism are as follows: TSH 0.4–4.0 mIU/L, free T3 2.5–6.0 pmol/L, free T4 10–24 pmol/L, and free T3:T4 0.1–0.6. *N* = 37 unless specified otherwise.

Abbreviations: TSH, thyroid stimulating hormone; Tg, thyroglobulin, T3,triiodothyronine; T4, thyroxine.

^a^ Thyroid peroxidase antibody (TPOAb) ≥10 IU/mL indicates positive.

^b^ Subclinical hypothyroidism: TSH >4.0 mIU/L and free T4 between 10 and 24 pmol/L.

^c^ Overt hypothyroidism: TSH >4.0 mIU/L and free T4 <10 pmol/L.

^d^ Subclinical hyperthyroidism: TSH <0.4 mIU/L and free T4 between 10 and 24 pmol/L.

^e^ Overt hyperthyroidism: TSH <0.4 mIU/L, free T4 >24 pmol/L, and free T3 >6.0 pmol/L.

^f^ Tg normal range: 3–40 μg/L, although >10 μg/L has been suggested to indicate iodine deficiency.^20^

Multiple linear regression including both plasma selenium and urinary iodine excretion as predictors of T3:T4 ratio was significant (*p* = 0.038) for predicting 13% of the model (adjusted *R* ^2^ = 0.127). Plasma selenium predicted T3:T4 ratio (*b* = 0.349, *p* = 0.033), although UIC was not significant (*b* = 0.287, *p* = 0.077; Table 5).

**TABLE 5:** Linear regression of plasma selenium and 24‐h urinary iodine excretion with T3:T4 ratio in midlife women (*n* = 37).

	*B*	SE *B*	*B*	*p*
Constant	0.253	0.051		
Iodine (UIE)	0.000	0.000	0.287	0.077
Plasma Se	0.008	0.004	0.349	0.033*

Abbreviations: Se, selenium; T3, triiodothyronine; T4, thyroxine; UIE, urinary iodine excretion.

* Statistically significant (<0.05).

## 4 DISCUSSION

Despite New Zealand government initiatives to improve iodine intake, this study found iodine deficiency among a group of midlife women with low bread intakes. The mUIC was 49 μg/L below both the WHO cut‐off (100 μg/L) and the alternative cut‐off for adults (60–70 μg/L).^21^ The 2014–2015 New Zealand Health Survey indicated iodine sufficiency in the population with mUIC of 91 μg/L among women.^26^ However, the current research suggests that iodine sufficiency may not be universal throughout the New Zealand population.

The EAR cut‐point method was used to determine the adequacy of population intake, where the percentage below the EAR approximates the proportion who are deficient.^27^ Using this definition, the current study participants had inadequate intake with the estimated median iodine intake from the 3‐day diet diaries at 63 μg/day and urinary excretion at 120 μg/day, with 82% and 41% having intakes below the EAR (100 μg/day), respectively. These current findings are lower than those found by Brough et al. in a study of healthy New Zealand women aged 50–70 years (*n* = 97) where median iodine intake was estimated using a 3‐day diet diary (138 μg/day) and urinary iodine (79 μg/day) and women reported consuming a median intake of 1.8 slices of bread per day.^17^ This current finding is not unexpected; however, it is concerning, suggesting those who do not consume bread could be at risk of iodine deficiency.

Median selenium intake estimated from 3‐day diet diaries and urinary excretion were 52 and 51 μg/day respectively, with 40% and 49% below the EAR (50 μg/day), indicating dietary inadequacy. Brough et al.^17^ found similar median selenium intakes based on a 3‐day diet diary (45 μg/day) and urinary excretion (50 μg/day) with 53% and 48% below the EAR, respectively. However, these values are lower than the most recent 2016 NZTDS which estimated average intakes among adult women at 68 μg/day, suggesting dietary inadequacy for some women in the study.^15^ The NZTDS data were generated from simulated diets from dietary data collected over a decade ago, rather than actual current diets. The average female adult in New Zealand would typically consume more bread than the participants of our study who consumed on average 1.6 serves of bread per week.

There is currently controversy over what is deemed an adequate selenium status, and the different selenoproteins have differing selenium concentrations required for function. In the present study, the median plasma selenium concentration was 118.9 μg/L, with all participants above 65–71 μg/L, suggested to be needed for optimal deiodinase function.^22^, ^23^ However, three participants had plasma concentrations below 95 μg/L, required for saturation of GPx activity.^24^ Furthermore, 12 participants (32%) had concentrations below 110 μg/L, which is recommended for maximum expression of selenoprotein P^25^ suggesting suboptimal status for some participants. A recent study of postpartum New Zealand women (*n* = 74) found a lower median plasma selenium concentration (105.8 μg/L) than in the current study; all participants achieved >65 μg/L, but 23% were less than 95 μg/L and 41% were below 110 μg/L.^28^ Equations have been derived that estimate selenium intake from plasma selenium concentrations; however, these are population specific and can be affected by the type of selenium consumed, so may not be universally applicable.^29^

A 1994 study in New Zealand found a much lower mean plasma selenium concentration of 85 μg/L in the Waikato and Taranaki regions.^8^ These regions are also in the North Island of New Zealand where bread is commonly made using selenium‐rich Australian wheat, and participants of this study were not excluded based on bread consumption. It might be expected that plasma concentrations in this population group would be higher than those of the current study where bread was being avoided, but this was many years ago and dietary habits may have changed. It is concerning that 32% of current study participants, who have low bread intakes, have sub‐optimal selenium status.

Although the current study participants had low bread intakes, some still had adequate intakes of selenium and iodine. Dairy was the greatest contributor to iodine, followed by fish and seafood, eggs and then bread. In the most recent NZTDS (2016) the highest contributors to iodine for adult females were grains, ‘chicken, eggs, fish and meat’, dairy, and ‘additional meat and shellfish’.^15^ This is similar to the current study, noting that even with such a low bread intake, bread was still the fourth greatest contributor of iodine due to its fortification.

For selenium intake the top four contributors for the present study were fish and seafood, nuts and seeds, meat and grains (excluding bread) which is similar to the 2016 NZTDS where the largest contributors for adult women were ‘chicken, eggs, fish and meat’ and grains.^15^ It is important to differentiate between the percentage a food contributes to selenium intake and the percentage it contributes to overall energy intake. New Zealand seafood has higher levels of selenium than other food sources^30^ and therefore a smaller quantity of seafood could be eaten to consume the same selenium content as a larger quantity of meat or poultry. Globally the richest sources of selenium are organ meats, Brazil nuts and some seafoods,^31^ thus, it is not surprising that fish and seafood, meat and nuts were the greatest contributors of selenium among these women with low bread intakes.

Although participants were recruited on the basis they consume less than one slice of bread per day, on average they were receiving 6.1% and 2.7% of total iodine and selenium intakes from bread and 4.5% and 9% from other grains (excluding bread), respectively. It is possible that intakes for selenium and iodine may have been lower if all bread and grain products were omitted from the diet. Some women were able to consume sufficient selenium and iodine even with low bread intakes; however, it is important to ensure alternative sources of both minerals are present in the daily diet to maintain adequate intake.

Interestingly, although the women had low bread and carbohydrate intakes, the mean fibre intake (23.9 g/day) was higher than the previous national survey of adult women (17.9 g/day),^11^ although still below the recommended Suggested Dietary Target (28 g/day).^13^ The main sources of fibre for the New Zealand population are bread, vegetables and potatoes, kumara and taro, fruit, grains and pasta, and breakfast cereals.^11^ Even though women in the current study are not getting much fibre from bread, they are still able to obtain it from other sources.

Most participants (92%) in the present study had euthyroidism. Only two participants were considered to have abnormal thyroid function, indicating subclinical hyperthyroidism. Four participants had a TPOAb result >10, indicating a potential autoimmune disorder. Previous studies have found selenium deficiency increases T4 concentrations due to the conversion of T4 to T3 requiring selenoprotein deiodinases^22^ and hence a decrease in the T3:T4 ratio. Median free T4 concentrations in this present study were 12.5 pmol/L, and all participants had concentrations within the reference range of 10–24 pmol/L. However, linear regression found that plasma selenium was a predictor of the T3:T4 ratio (*p* = 0.033). This could indicate selenium deficiency in some of the current study population as selenium status decreases; T4 increases. All current participants had serum plasma above the cut‐off for deiodinases of 65–71 μg/L, and yet selenium status was still associated with the T3:T4 ratio, suggesting a higher plasma selenium concentration may be required for optimal T4 to T3 conversion. The lack of association with UIE is not surprising as a single measure for UIC is not a valid measure for individual status; at least 10 measures are necessary to determine an individual's iodine status.^32^ No other selenium or iodine measures (status or intake) were associated with thyroid hormone concentrations.

To our knowledge, this was the first study to investigate the effect of low bread intake on iodine and selenium status and thyroid function in New Zealand women. The strengths of this study are the use of robust dietary and biological markers to estimate iodine and selenium intake and status, together with the assessment of thyroid function. Selenium status was measured using plasma concentrations as this is a useful biomarker at an individual and population level.^33^ However, plasma and serum concentrations only reflect short‐term selenium intake (several days) and it may be beneficial to measure a functional marker of selenium status, such as GPx activity or selenoprotein P.^24^, ^25^ Other limitations of this study include the small sample size and use of one 3‐day diet diaries from April to August (autumn and winter in New Zealand) which may not be representative of ‘usual intake’. As participants are from the Auckland region, this sample may not represent the wider New Zealand population, especially the South Island, where low intakes of bread could have less effect on selenium intakes.

Overall, a high prevalence of inadequate iodine and selenium intake was observed in this group of mid‐life New Zealand women who avoided bread. Urinary iodine showed the group to be iodine deficient, and almost a third of participants had suboptimal selenium plasma concentrations. Plasma selenium concentrations predicted the ratio of T3:T4, highlighting the role of selenium in thyroid function. It is possible to meet selenium and iodine requirements without consuming bread; however, care must be taken to ensure alternative sources of both minerals are consumed, such as dairy, fish and seafood, eggs, meat, other grains, and nuts. This study highlights the unconsidered consequences of eliminating a food group from the diet without a full understanding of the nutrient contributions from those foods.

There has been no national dietary survey in New Zealand since 2009. Although the current study is only of low bread consumers, we do not know how widespread this dietary pattern is. Dairy is also a good source of iodine; however, in recent years, plant milks consumption has increased, but we do not know if this is impacting iodine status. Larger studies are required to investigate current dietary trends within New Zealand and any adverse effects on selenium and iodine status and thyroid function. Alternative strategies may be required to ensure the whole New Zealand population has adequate iodine and selenium intake, such as raising awareness of the best dietary sources among at‐risk groups or consideration of the fortification of other foods.

## AUTHOR CONTRIBUTIONS

All authors contributed to the study conception and design. Recruitment and data collection was performed by Jacqueline Finlayson. Data analyses were performed by Jaime Berger, Jacqueline Finlayson, and Louise Brough. The first draft of the manuscript was written by Jaime Berger and all authors commented on previous versions of the manuscript. All authors read and approved the final manuscript.

## FUNDING INFORMATION

This research was supported with funding from the School of Sport, Exercise and Nutrition, College of Health, Massey University, New Zealand.

## CONFLICT OF INTEREST STATEMENT

The authors declare no conflicts of interest.

## Data Availability

The datasets generated during and/or analysed during the current study are not publicly available due to privacy and ethical restrictions.

## References

[1] Schomburg L , Kohrle J . On the importance of selenium and iodine metabolism for thyroid hormone biosynthesis and human health. Mol Nutr Food Res. 2008;52:1235‐1246. 18686295 10.1002/mnfr.200700465

[2] Zimmermann MB , Jooste PL , Pandav CS . Iodine‐deficiency disorders. Lancet. 2008;372:1251‐1262. 18676011 10.1016/S0140-6736(08)61005-3

[3] Zimmermann MB , Köhrle J . The impact of iron and selenium deficiencies on iodine and thyroid metabolism: biochemistry and relevance to public health. Thyroid. 2002;12:867‐878. 12487769 10.1089/105072502761016494

[4] Rayman MP . Selenium and human health. Lancet. 2012;379:1256‐1268. 22381456 10.1016/S0140-6736(11)61452-9

[5] Rohner F , Zimmermann M , Jooste P , et al. Biomarkers of nutrition for development—iodine review. J Nutr. 2014;144:1322S‐1342S. 24966410 10.3945/jn.113.181974 PMC4093988

[6] Ministry of Health . A Portrait of Health: Key Results of the 2006/07 New Zealand Health Survey. Ministry of Health; 2008.

[7] Gibbons V , Conaglen JV , Lillis S , Naras V , Lawrenson R . Epidemiology of thyroid disease in Hamilton (New Zealand) general practice. Aust N Z J Public Health. 2008;32:421‐423. 18959544 10.1111/j.1753-6405.2008.00273.x

[8] Thomson CD . Selenium and iodine intakes and status in New Zealand and Australia. Br J Nutr. 2004;91:661‐672. 15137917 10.1079/BJN20041110

[9] Mann JI , Aitken E . The re‐emergence of iodine deficiency in New Zealand? N Z Med J. 2003;116:U351. 12658310

[10] Food Standards Australia New Zealand . Australia New Zealand Food Standards Code, Standard 2.1.1. Cereals and Cereal Products. Accessed September 5, 2024. https://www.legislation.gov.au/F2015L00420/latest/text

[11] Ministry of Health . A Focus on Nutrition: Key Findings of the 2008/09 New Zealand Adult Nutrition Survey. Ministry of Health; 2011.

[12] Thomson CD . Assessment of requirements for selenium and adequacy of selenium status: a review. Eur J Clin Nutr. 2004;58:391‐402. 14985676 10.1038/sj.ejcn.1601800

[13] National Health and Medical Research Council, New Zealand Ministry of Health . Nutrient Reference Values for Australia and New Zealand Including Recommended Dietary Intakes. National Health and Medical Research Council; 2006.

[14] Ministry of Agriculture and Forestry . 2009 New Zealand Total Diet Survey—Agricultural Compound Residues, Selected Contaminant and Nutrient Elements. Ministry of Agriculture and Forestry; 2011.

[15] Ministry for Primary Industries . 2016 New Zealand Total Diet Survey. Ministry for Primary Industries; 2018.

[16] Mallard SR , Gray AR , Houghton LA . Periconceptional bread intakes indicate New Zealand's proposed mandatory folic acid fortification program may be outdated: results from a postpartum survey. BMC Pregnancy Childbirth. 2012;12:8. 22333513 10.1186/1471-2393-12-8 PMC3305569

[17] Brough L , Gunn CA , Weber JL , et al. Iodine and selenium intakes of postmenopausal women in New Zealand. Nutrients. 2017;9(3):254. 10.3390/nu9030254 28282932 PMC5372917

[18] Ministry for Primary Industries . Update Report on the Dietary Iodine Intake of New Zealand Children Following Fortification of Bread with Iodine. Ministry for Primary Industries; 2014.

[19] Edmonds JC , McLean RM , Williams SM , Skeaff SA . Urinary iodine concentration of New Zealand adults improves with mandatory fortification of bread with iodised salt but not to predicted levels. Eur J Nutr. 2016;55:1201‐1212. 26018655 10.1007/s00394-015-0933-y

[20] Zimmermann MB . Methods to assess iron and iodine status. Br J Nutr. 2008;99(suppl 3):S2‐S9. 10.1017/S000711450800679X 18598585

[21] Zimmermann MB , Andersson M . Assessment of iodine nutrition in populations: past, present, and future. Nutr Rev. 2012;70:553‐570. 23035804 10.1111/j.1753-4887.2012.00528.x

[22] Thomson CD , McLachlan SK , Grant AM , Paterson E , Lillico AJ . The effect of selenium on thyroid status in a population with marginal selenium and iodine status. Br J Nutr. 2005;94:962‐968. 16351774 10.1079/bjn20051564

[23] Winther KH , Rayman MP , Bonnema SJ , Hegedüs L . Selenium in thyroid disorders—essential knowledge for clinicians. Nat Rev Endocrinol. 2020;16:165‐176. 32001830 10.1038/s41574-019-0311-6

[24] Thomson CD , Robinson MF , Butler JA , Whanger PD . Long‐term supplementation with selenate and selenomethionine: selenium and glutathione peroxidase (EC 1.11.1.9) in blood components of New Zealand women. Br J Nutr. 1993;69:577‐588. 8490010 10.1079/bjn19930057

[25] Hurst R , Armah CN , Dainty JR , et al. Establishing optimal selenium status: results of a randomized, double‐blind, placebo‐controlled trial. Am J Clin Nutr. 2010;91:923‐931. 20181815 10.3945/ajcn.2009.28169 PMC2844680

[26] Ministry for Primary Industries . Mandatory Iodine Fortification in New Zealand: Supplement to the Australian Institute of Health and Welfare 2016 Report—Monitoring the Health Impacts of Mandatory Folic Acid and Iodine Fortificaton. Ministry for Primary Industries and Ministry of Health; 2016.

[27] Institute of Medicine . Dietary Reference Intakes: Applications in Dietary Assessment. National Academy Press; 2000.

[28] Jin Y , Coad J , Pond R , Kim N , Brough L . Selenium intake and status of postpartum women and postnatal depression during the first year after childbirth in New Zealand—Mother and Infant Nutrition Investigation (MINI) study. J Trace Elem Med Biol. 2020;61:126503. 32442890 10.1016/j.jtemb.2020.126503

[29] EFSA Panel on Nutrition NF , Allergens F , Turck D , et al. Scientific opinion on the tolerable upper intake level for selenium. EFSA J. 2023;21:e07704. 36698500 10.2903/j.efsa.2023.7704 PMC9854220

[30] Thomson BM , Vannoort RW , Haslemore RM . Dietary exposure and trends of exposure to nutrient elements iodine, iron, selenium and sodium from the 2003‐4 New Zealand total diet survey. Br J Nutr. 2008;99:614‐625. 17925056 10.1017/S0007114507812001

[31] Rayman MP . Food‐chain selenium and human health: emphasis on intake. Br J Nutr. 2008;100:254‐268. 18346308 10.1017/S0007114508939830

[32] Andersen S , Karmisholt J , Pedersen KM , Laurberg P . Reliability of studies of iodine intake and recommendations for number of samples in groups and in individuals. Br J Nutr. 2008;99:813‐818. 17961291 10.1017/S0007114507842292

[33] Phiri FP , Ander EL , Lark RM , et al. Urine selenium concentration is a useful biomarker for assessing population level selenium status. Environ Int. 2020;134:105218. 31715489 10.1016/j.envint.2019.105218

